# Augmented Inhibition from Cannabinoid-Sensitive Interneurons Diminishes CA1 Output after Traumatic Brain Injury

**DOI:** 10.3389/fncel.2014.00435

**Published:** 2014-12-19

**Authors:** Brian N. Johnson, Chris P. Palmer, Elliot B. Bourgeois, Jaclynn A. Elkind, Brendan J. Putnam, Akiva S. Cohen

**Affiliations:** ^1^Children’s Hospital of Philadelphia Research Institute, Children’s Hospital of Philadelphia, Philadelphia, PA, USA; ^2^Department of Neuroscience, University of Pennsylvania School of Medicine, Philadelphia, PA, USA; ^3^Department of Pathology, Brigham and Women’s Hospital, Harvard Medical School, Boston, MA, USA; ^4^Department of Pediatrics, University of Pennsylvania School of Medicine, Philadelphia, PA, USA

**Keywords:** interneuron, traumatic brain injury, CA1, cholecystokinin, cannabinoid type 1 receptor, action potential, lateral fluid percussion, voltage-sensitive dye

## Abstract

The neurological impairments associated with traumatic brain injury include learning and memory deficits and increased risk of seizures. The hippocampus is critically involved in both of these phenomena and highly susceptible to damage by traumatic brain injury. To examine network activity in the hippocampal CA1 region after lateral fluid percussion injury, we used a combination of voltage-sensitive dye, field potential, and patch clamp recording in mouse hippocampal brain slices. When the stratum radiatum (SR) was stimulated in slices from injured mice, we found decreased depolarization in SR and increased hyperpolarization in stratum oriens (SO), together with a decrease in the percentage of pyramidal neurons firing stimulus-evoked action potentials. Increased hyperpolarization in SO persisted when glutamatergic transmission was blocked. However, we found no changes in SO responses when the alveus was stimulated to directly activate SO. These results suggest that the increased SO hyperpolarization evoked by SR stimulation was mediated by interneurons that have cell bodies and/or axons in SR, and form synapses in stratum pyramidale and SO. A low concentration (100 nM) of the synthetic cannabinoid WIN55,212-2, restored CA1 output in slices from injured animals. These findings support the hypothesis that increased GABAergic signaling by cannabinoid-sensitive interneurons contributes to the reduced CA1 output following traumatic brain injury.

## Introduction

A full understanding of the macrocircuits that ultimately underlie brain function and behavior requires a complete understanding of the microcircuits upon which they are built. The response of the hippocampus to lateral fluid percussion injury (lFPI), a well-characterized mouse model of traumatic brain injury (TBI; Thompson et al., 2005; Xiong et al., 2013), highlights the need for detailed information regarding such circuits, as lFPI exerts opposing effects on the dentate gyrus and the CA1 region (Witgen et al., 2005; Schwarzbach et al., 2006; Cole et al., 2010). Network excitability increases in the dentate gyrus, but decreases in CA1. Regional network excitability depends on the intrinsic properties of individual cells (e.g., resting membrane potential, input resistance, and action potential threshold) as well as the strength and type (e.g., excitatory or inhibitory) of the synaptic connections between cells.

**Figure 1:**
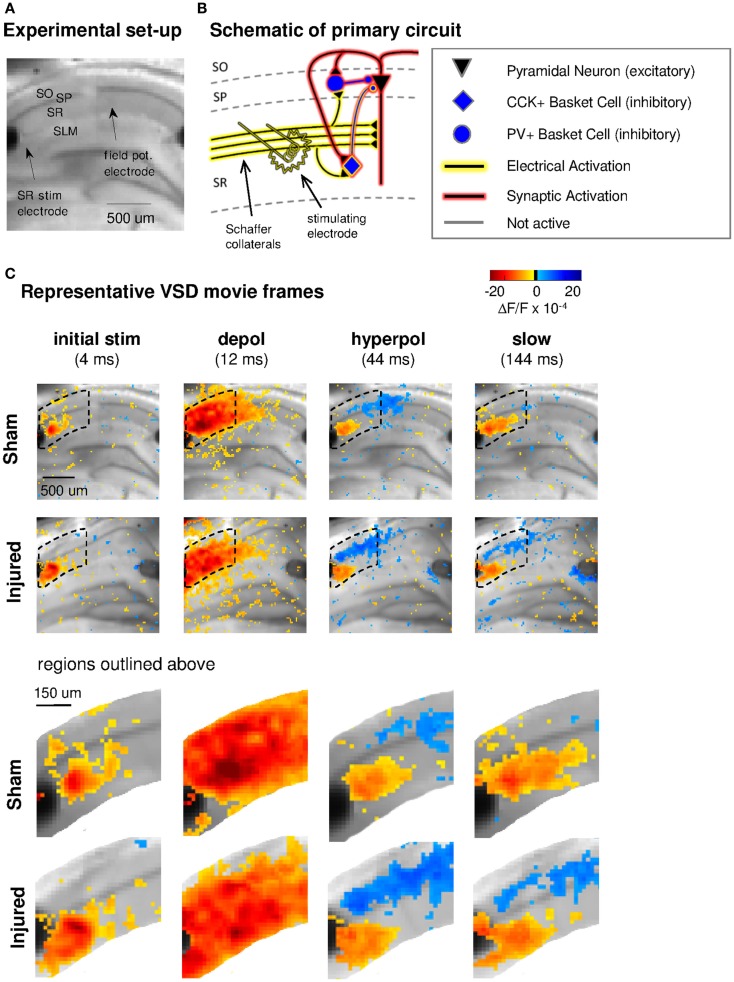
**Experimental set-up, schematic diagram of relevant circuitry, and movie frames from representative VSD recordings**. **(A)** Representative sham slice photomicrograph showing locations of electrodes and cell layers. SR stimulating electrode is labeled at left, SLM stimulating is visible at right but unlabeled. Arrows indicate approximate location of tips of stimulating and recording electrodes. Light gray spot to left of arrow marking field potential electrode, and partially obscuring cell body layer, is meniscus around field potential electrode where it penetrated the surface of the solution. In this and subsequent figures, data are arranged in the order of the cell layers shown here, with SO uppermost, then SP and SR and (when shown) SLM. **(B)** Schematic of CA1 pyramidal neuron and basket cell activation by SR stimulation in aCSF. Note distinction between direct “electrical activation” shown by yellow highlight, and “synaptic activation” shown by red highlight. “Electrical activation” refers to the direct depolarization of cell membranes by the current flow from a stimulating electrode, independent of any subsequent synaptic depolarization. “Synaptic activation” refers to depolarization initiated by synaptic currents. Legend here applies also to schematics in subsequent figures. **(C)** Representative movie frames from VSD recordings of hippocampal brain slices from sham and injured mice. Warmer colors (red, orange, and yellow) indicate depolarization (positive ΔF/F values), cooler colors (light blue and dark blue) indicate hyperpolarization (negative ΔF/F values). The initial response (4 ms post-stimulus) is depolarization proximal to the stimulating electrode (yellow to orange pixels, 3–13 × 10^−4^ ΔF/F), followed in sequence by stronger and more widespread depolarization (12 ms, yellow to red pixels, 3–28 × 10^−4^ ΔF/F), then a drop to less depolarized voltages in SR (44 ms, 0–10 × 10^−4^ ΔF/F) paired with net hyperpolarization in SO (0 to −7 × 10^−4^ ΔF/F), and finally a slow return to pre-stimulus levels. Regions inside dotted outline are expanded in lower panels. After injury, hyperpolarization in SO is larger (more dark blue pixels at 44 ms). Slow response in slice from injured animal also shows a shift toward more hyperpolarized levels in both SR and SO. Stimulus: 100 μA. Color scale bar blackened region (centered around zero) denotes noise threshold (2 SD of the fluorescence signal during the 64 ms pre-stimulus period) used for pseudocoloring the movie frames. SO, stratum oriens; SP, stratum pyramidale; SR, stratum radiatum; SLM, stratum lacunosum-moleculare; CCK, cholecystokinin; PV, parvalbumin; stim, stimulus; depol, depolarization; hyperpol, hyperpolarization. Stimulating electrodes are visible at left (SR) and right (SLM) edges of full frame images, although only the SR electrode was used for the experiments in this figure.

A brief review of those connections and hippocampal anatomy in general, helps to frame our results. The dentate gyrus is the primary input region of the hippocampus, while CA1 is the primary output region. Afferents from the granule cells in the DG project to pyramidal neurons in the CA3 region, which in turn project to pyramidal neurons in CA1. The afferents from those CA3 pyramidal neurons are known as the Schaffer Collateral pathway (Figures 1A,B). Another feature of hippocampal anatomy is its laminar organization. In CA1, the pyramidal neuron cell bodies constitute the stratum pyramidale (SP). The stratum radiatum (SR) contains the proximal portion of the CA1 pyramidal neuron apical dendrites as well as the Schaffer collaterals, which form synapses on those apical dendrites. The stratum lacunosum-moleculare (SLM) contains the distal portion of the dendrites, and receives inputs from the entorhinal cortex. The basal dendrites of the CA1 pyramidal neurons extend into the stratum oriens (SO), which also contains the CA1 pyramidal neuron axons, which radiate outward and enter the alveus.

All of the layers in CA1 also contain local inhibitory interneurons, and much of the circuit complexity in CA1 is attributable to the many different kinds of interneurons in this region, each of which has a distinct neurochemical and anatomical profile, and is thought to serve a different role in modulating network function (Klausberger and Somogyi, 2008). Basket cell interneurons, for instance, provide the majority of perisomatic inhibition to pyramidal neurons, and modulate action potential firing (Miles et al., 1996; Freund and Katona, 2007). Basket cells are a fundamental cell type in the hippocampus and cortex, and can be divided into two independent subgroups according to the mutually exclusive presence of parvalbumin (PV) or cholecystokinin (CCK). PV containing basket cells are instrumental in establishing the gamma frequency network oscillations essential to normal cognitive function, while CCK positive basket cells are thought to provide modulatory control of those oscillations (Freund, 2003). We previously reported an increase in action potential-independent CA1 miniature inhibitory postsynaptic current amplitude following lFPI and a decreased ratio of the somatic population spike to the dendritic excitatory postsynaptic potential (EPSP) (Witgen et al., 2005), the latter of which is a measure of the coupling between the excitatory synaptic input to CA1 pyramidal cells and their collective action potential output. Both of these findings are consistent with an increase in stimulus-evoked inhibition after lFPI. Moreover, the inhibitory GABA_A_ receptor antagonist bicuculline eliminated the difference in evoked dendritic field potential responses, suggesting that the decreased excitability in CA1 after injury was due primarily to augmented inhibition. The methodology used in those earlier studies, however, did not allow us to determine the population of interneurons affected or the site of expression.

Voltage-sensitive dye (VSD) imaging can simultaneously record the activity of hundreds to thousands of neurons and is well-suited for evaluating inhibitory circuitry, as it can directly monitor regional hyperpolarization. In the current study, we used a combination of VSD imaging, field potentials, and whole-cell current clamp recordings to explore normal and injury-altered CA1 network function. We found increased hyperpolarization in SO after injury and a decrease in CA1 output as measured by the percentage of pyramidal neurons firing stimulus-evoked action potentials (AP). This decrease in action potential firing was eliminated by a low concentration (100 nM) of the CB1 receptor agonist WIN55,212-2, which at this concentration did not significantly affect glutamatergic transmission. These results suggest that augmented inhibition from cannabinoid-sensitive interneurons contributes to the reduced CA1 output after injury.

## Materials and Methods

### Lateral fluid percussion injury

All experiments were performed on 6- to 10-week-old male C57/BL6 mice (Jackson Laboratory, Bar Harbor, ME, USA). lFPI is a well-characterized model that mimics human TBI pathology (Thompson et al., 2005; Xiong et al., 2013). Briefly (see Witgen et al. (2005) for full details) mice were anesthetized with ketamine/xylazine and after performing a craniectomy, a Luer-loc needle hub (3 mm inner diameter) was secured above the skull opening. The next day, the mouse was anesthetized with isoflurane and the hub was filled with saline and connected via high-pressure tubing to the lFPI device (Department of Biomedical Engineering, Virginia Commonwealth University, Richmond, VA, USA). Injury was induced by a 10–15 ms pulse of saline (1.5–1.8 atm peak pressure, monitored with a pressure transducer attached to an oscilloscope) onto the intact dura creating both a local and diffuse injury via the propagation of a pressure wave through the intact brain. Sham animals received all of the above except the fluid pulse. Subsequent experiments were performed 6–8 days after injury. There were no differences in gross hippocampal anatomy between slices prepared from sham and injured animals. Mice were observed daily for any discomfort associated with lFPI or sham surgery and all procedures were approved by the Children’s Hospital of Philadelphia IACUC committee.

Lateral fluid percussion injury in our model is designed to produce a mild to moderate non-penetrating (dura is not breached) brain injury. The righting time reflex, i.e., the length of time after the injury until the animal spontaneously righted itself, was used as an acute neurological assessment of the severity of the injury (Morehead et al., 1994). Animals with an excessive righting time, indicating a more severe injury, were excluded from the study. Blood gases and arterial pressure were not recorded due to technical limitations of the mouse model, and because they are thought to be more relevant to severe brain injury, than to the mild to moderate brain injury delivered here. lFPI in our model leads to hippocampal-dependent anterograde and retrograde cognitive impairment as demonstrated by the decreased freezing exhibited by lFPI animals in contextual fear conditioning studies (Witgen et al., 2005; Lifshitz et al., 2007; Cole et al., 2010). Furthermore, lFPI produces an inability to maintain wakefulness including a significant decrease in theta oscillations during rapid eye movement sleep (Lim et al., 2013). In addition, lFPI leads to a significant reduction (30–35%) in the number of neurons in all subregions of the ipsilateral hippocampus (CA1, CA3, hilus and dentate gyrus) as measured using unbiased stereology (Witgen et al., 2005).

### Electrophysiology

**Figure 2:**
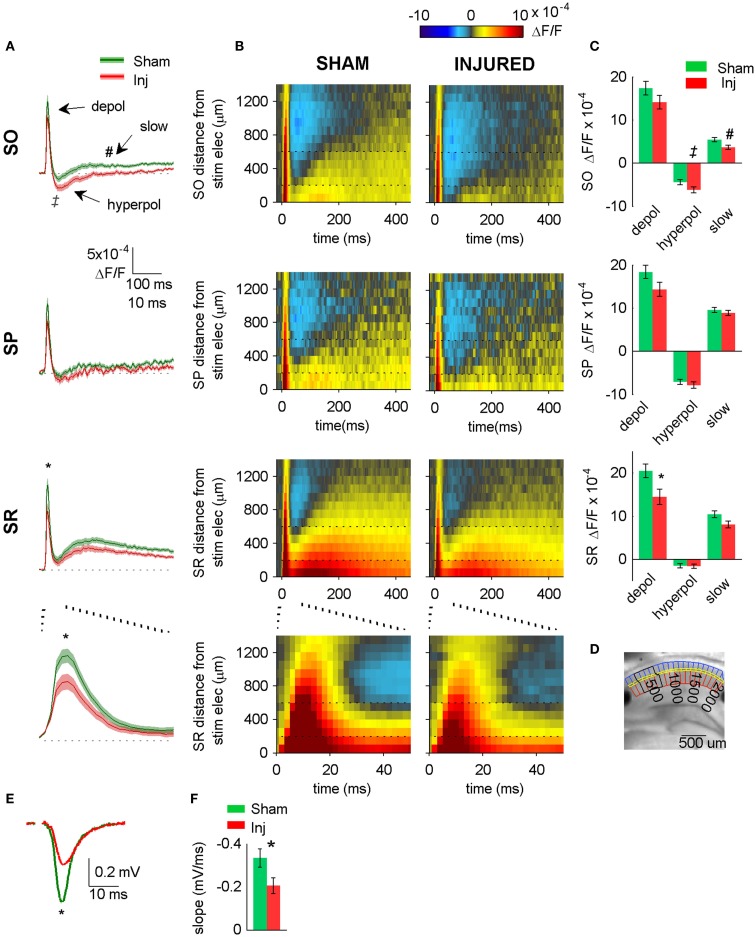
**Hyperpolarizing shift in SR-evoked responses after injury**. **(A)** Group average VSD signal shows decreased depolarization in SR (lower two panels), increased hyperpolarization in SO (upper panel) after injury. Average traces for sham (*n* = 25) and injured (*n* = 22) calculated from region indicated in **(D)** below. Shaded region for each plot in this and subsequent figures indicates mean ± SE. Bottom panel shows lower middle panel on expanded time scale. **(B)** Group average raster representation of sham (*n* = 25) and injured (*n* = 22) VSD responses. Each point in the raster shows the average fluorescence signal, ΔF/F, for the segment at the indicated distance from stimulating electrode, and at the indicated time. A row shows all time points for a given segment; a column shows all segments at a given time. Note decreased depolarization in SR (fewer red pixels, middle and lower panels), increased hyperpolarization in SO (more blue pixels, upper panels). Bottom panels show middle panels on expanded time scale. Note increased hyperpolarization in SO (more blue pixels starting at approximately 20 ms). Horizontal dotted lines indicate raster region corresponding to multi-segment analysis region (segments 3–6). Bottom panels show lower middle panels on expanded time scale. **(C)** Group data showing significant hyperpolarizing shift in fast depolarization in SR and hyperpolarization in SO. Simultaneous field potential recordings in SR were also significantly smaller after injury **(E)**. **(D)** Representative slice showing segmentation used for rasters (SO segments in blue, SP segments in yellow, SR segments in red), and region used to calculate average traces and perform statistics (black outline around segments 3–6). The symbols * and ‡indicate significant differences (*P* < 0.05) in the fast depolarization and hyperpolarization respectively. **(E)** Representative field potentials from two individual slices recorded during VSD trials (SR stimulation and recording). The field potential from the representative injured animal slice is smaller than the field potential from the representative sham animal slice. **(F)** Group average statistics for field potentials recorded during VSD trials indicate a significant decrease after injury (sham −0.334 ± 0.043 mV/ms, *n* = 23; injured −0.206 ± 0.036 mV/ms, *n* = 22; *P* = 0.025). Asterisks denote significant difference in **(E,F)**. SO, stratum oriens; SP, stratum pyramidale; SR stratum radiatum; Inj, Injured. Stimulus: 100 μA for both VSD and field potential recordings.

**Figure 3:**
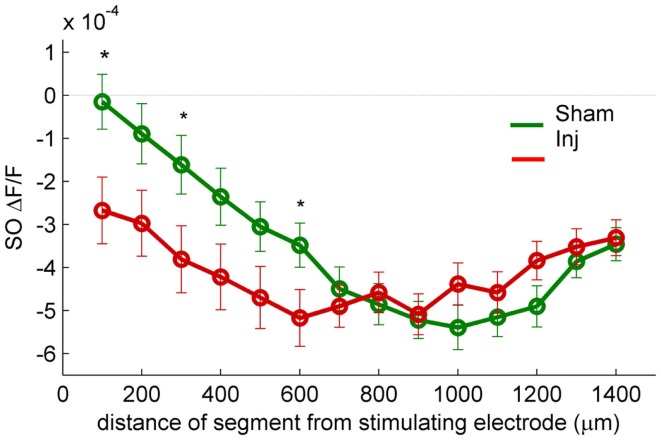
**SR-evoked SO hyperpolarization is larger near the stimulating electrode in slices prepared from injured mice**. VSD single segment average SR-evoked hyperpolarization in SO and proximal SP as a function of distance from the stimulating electrode. Asterisks indicate significant difference between sham and injured slices in the indicated segments (Mann–Whitney *P* < 0.05). Individual segments 1, 3, and 6 were significantly different, as was the average of segments 1 through 6. None of the individual segments 7 or higher were significantly different between sham and injured. These results are consistent with the hypothesis that the increased SO hyperpolarization after injury is due to an increase in GABAergic signaling subsequent to the direct stimulation of interneuron cell bodies and/or axons near the stimulating electrode.

Mice were anesthetized with isoflurane, and their brains were quickly and carefully removed and placed into ice-cold oxygenated (95% O_2_/5% CO_2_) sucrose-containing artificial cerebrospinal fluid (aCSF) containing (in mM): sucrose 202, KCl 3, NaH_2_PO_4_ 2.5, NaHCO_3_ 26, glucose 10, MgCl_2_ 1, CaCl_2_ 2. Coronal slices 350 μm thick were cut on a vibratome (VT1200S, Leica Microsystems, Buffalo Grove, IL, USA) and transferred to 33–37°C oxygenated (95% O_2_/5% CO_2_) control aCSF containing (in millimolar): NaCl 130, KCl 3, NaH_2_PO_4_ 1.25, NaHCO_3_ 26, glucose 10, MgCl_2_, 1 CaCl_2_ 2. After 60–90 min slices were allowed to cool to room temperature. All VSD and field potential recordings were performed in an interface chamber with a flow rate of 1.5–2.0 ml/min, and kept at 27–30°C. Slices for sham and injured animals were selected from the same dorsal-ventral region of the hippocampus, typically 1.70–2.46 mm posterior to Bregma [e.g., Figures 45 through 51 in Paxinos and Franklin (2001)]. Extracellular field potential recording electrodes were fabricated from borosilicate glass (World Precision Instruments, Sarasota, FL, USA, #1B150F-4) pulled to a tip resistance of 2–6 MΩ and filled with aCSF. Field potentials were recorded in CA1 SR with an Axoclamp 900A amplifier and pClamp10 data acquisition software (Molecular Devices, Sunnyvale, CA, USA), and filtered at 2 kHz. Field potential stimulating electrodes were concentric and bipolar (Frederick Haer Corporation, Bowdoin, ME, USA, #CBDPG75). The SR stimulating electrode was placed near the CA1 border, and approximately two-thirds of the distance from the cell body layer to the boundary between SR and SLM, which in our slices was approximately 200 μm from the border of SP and SR (Figure 1A). The SR field potential recording electrode was placed approximately 900–1100 μm distal (along the cell body layer) to the stimulating electrode and two-thirds of the distance from the cell body layer to the boundary between SR and SLM, which again was approximately 200 μm from the border between SP and SR (Figure 1A). Recording and stimulating electrodes were iteratively lowered to the depth that gave the maximum response. Electrical stimuli were 100 μs in duration. Field potentials were recorded prior to all VSD experiments with single stimuli ranging from 50 to 750 μA in 50 μA increments, and also as 10 trial averages at 300 μA, which was the approximate half-maximal response level. The inter-stimulus interval for these field potentials was 8 s. Ten pairs of stimuli were also delivered with a paired pulse inter-stimulus interval of 75 ms, and an inter-pair interval of 8 s. In addition, field potentials were recorded during the VSD imaging trials, in response to the same electrical stimuli used to produce stimulus-evoked VSD responses. The inter-stimulus interval for field potentials recorded during the VSD recordings was 20 s, since non-electrically stimulated VSD trials were interleaved with electrically stimulated VSD trials (see “VSD imaging” below). We did not observe any changes in the amplitude of these single stimulus responses consequent to repeated stimulation. To keep the experiments manageably short and limit response instability, the 26-trial VSD recordings were typically performed at no more than two or three different stimulus strengths. As shown in the VSD recordings below (Figures 1–3), even comparatively small extracellular stimuli (e.g., 100 μA, which was below the half-maximal response level for simultaneous field potentials) activate a surprisingly large region of tissue, and so our general approach was to use the smallest stimuli possible, which still produced a large signal-to-noise ratio. In practice, for a given test condition 100 μA stimuli were used for all slices in both sham and injured groups if 100 μA stimuli produced easily detectable responses in all slices in both sham and injured groups (e.g., the control aCSF response to SR stimulation); otherwise 300 μA stimuli were used for all the slices in both the sham and injured groups (e.g., the responses in solution containing APV and CNQX). The stimulus used for a given test condition is specified in the corresponding figure legend. Patch-electrode whole-cell current clamp recordings were performed in a submersion chamber, with a flow rate of 1.0–1.5 ml/min, at 22–24°C. Patch electrodes for the whole-cell current clamp recordings were fabricated from borosilicate glass (1B150F-4, World Precision Instruments, Sarasota, FL, USA) pulled to a tip resistance of 3–7 MΩ and filled with internal solution containing (in mM): KGluconate 145, KCl 2.5, NaCl 2.5, HEPES 10, MgCl_2_ 2, ATP.Mg 2, and GTP.Tris 0.5. Slices were kept at 33–37°C prior to recording. All patch-electrode current clamp recording voltages were corrected for a liquid junction potential calculated at −14.5 mV (using the Junction Potential utility in Clampex 10.3, Molecular Devices, Sunnyvale, CA, USA), and a series voltage error of 2.2 mV. Resting membrane potentials were measured shortly after break-in, and then current was injected to maintain cells near the target resting potential of −76 mV. During current clamp recordings, cells were periodically switched to voltage clamp long enough to measure series resistance. At the conclusion of the current clamp recordings the electrode was withdrawn from the cell, and any potential measured in the extracellular space was subtracted from the voltages recorded for that cell. Whole-cell current clamp stimulating electrodes were non-concentric bipolar (World Precision Instruments, Sarasota, FL, # ME12206), and placed in SR approximately 700–900 μm from the recording electrode; stimuli were 100 μs in duration and the same set of stimulus strengths (100–900 μA in 100 μA increments) was used for slices from both sham and injured animals.

### Voltage-sensitive dye imaging

Dye stock solutions of di-3-ANEPPDHQ (Invitrogen) were prepared at a concentration of 20 mg/ml in ethanol and stored at −20°C. Dye working solutions were prepared at a concentration of 67 μg/ml by diluting dye stock solution 1/300 in aCSF on the day of recording, and slices were stained for 16 min, after which they were rinsed thoroughly before being transferred to the recording chamber. Stained slices were then washed for another 15–20 min prior to commencing VSD recording. The dye was excited by seven high-power green LEDs (Luxeon Rebel LXML-PM01-0100, Philips) coupled to a 535 ± 25 nm bandpass filter and 565 nm dichroic mirror. Emitted VSD fluorescence was isolated with a 610 nm longpass filter and recorded at 0.5 kHz with a fast video camera with 80 × 80 pixel resolution (NeuroCCD, Redshirt Imaging, Decatur, GA, USA) through a reverse-lens macroscope with a 50 mm f/1.3 M46 lens (Dark Invader). Each camera pixel imaged a 25 μm × 25 μm region of tissue. The fluorescence light source was triggered 230 ms prior to the acquisition of fluorescence data to allow the light onset emission transient to stabilize, and the electrical stimulus was delivered 170 ms after commencing fluorescence data acquisition. Typical recordings were 1.0–1.5 s in duration. Imaging trials were separated by a 20-s interval. Electrically stimulated and non-electrically stimulated trials were interleaved for subsequent subtraction of background fluorescence. A total of 26 trials were performed for each experimental condition.

### Analysis

Field potential amplitudes were calculated as the slope of the initial linear portion of the response. In the whole-cell current clamp recordings, the reversal potential for GABA_A_ in our solutions was calculated to be −70.4 mV, assuming a bicarbonate to chloride permeability ratio of 0.2 according to Tyzio et al. (2008), and assuming an internal bicarbonate concentration less than or equal to 0.1 mM in our nominally bicarbonate free internal solution. Action potential thresholds were calculated as the voltage during an AP at which the slope of the voltage with respect to time first exceeded 30 mV/ms (Howard et al., 2007). As the AP thresholds and their value relative to the GABA_A_ receptor reversal potential (E_GABA_A__) are an important aspect of our data, we have taken care to correct all of our voltage measurements for the calculated liquid junction potential (see Methods), which was 14.5 mV in our potassium gluconate based internal. The AP thresholds reported here are in excellent agreement with literature reports of AP thresholds measured in mouse CA1 whole-cell current clamp recordings (Fink and O’Dell, 2009; Routh et al., 2009; Zhang et al., 2010; Brown et al., 2011; Minge and Bahring, 2011; Wykes et al., 2012), when the liquid junction potential is appropriately subtracted from the non-corrected voltages in those reports.

All VSD trials were screened manually, and any trials showing evidence of gross contamination from ambient light outside of the shaded recording enclosure (e.g., a large and continuous drift throughout the pre-stimulus period) were excluded from further analysis. Fractional change in fluorescence values (ΔF/F) were calculated as follows: (1) fluorescence values for each pixel in each trial were normalized according to the average fluorescence in the pixel during a 64 ms window immediately preceding the electrical stimulus, then (2) an average non-electrically stimulated trial was normalized and subtracted from the individual electrically stimulated trials to correct for photo-bleaching. VSD recordings were filtered in x and y spatial coordinates by convolution with a 5 pixels × 5 pixels Gaussian filter (sigma 1.2 pixels), and in time by convolution with a five sample median filter. No additional filtering was applied to any of the images, or regional average line plots. Raster representation of the VSD recordings was begun by drawing boundary lines around SO (blue segments in Figure 2D), SP (SP, yellow segments in Figure 2D), and SR (red segments in Figure 2D), then these regions were split into 100 μm wide segments, where the first segment contained the points 0–100 μm from the stimulating electrode. The average value of the fluorescence signal (ΔF/F) was calculated for each segment and plotted as pseudocolor in the distance from stimulating electrode versus time rasters, and as multi-segment regional averages in the line plots. All tests for statistical significance were Mann–Whitney *U*-tests, except where indicated otherwise. No differences were observed in responses from naïve versus sham-injured mice, therefore data from these animals were pooled for purposes of analysis. All bar graphs show mean ± SEM. For each test condition all graphical and statistical comparisons between sham and injured were made using the same stimulus strengths in both groups. VSD data was collected in 70 slices prepared from 39 animals. Whole-cell current clamp data was collected in 26 cells in 23 slices prepared from 16 animals.

### Terminology and analysis region specification

We refer to the location of the pathway stimulating electrodes by the anatomical lamina in which the electrodes are situated (e.g., SR) and not the nominal pathway (e.g., the Schaffer collaterals) to emphasize the simultaneous direct electrical stimulation of other circuit elements, e.g., interneuron cell bodies and axons, in addition to the nominal pathway axons. We defined three time intervals for statistical analysis of the VSD recordings. The “fast depolarization” (peak measurement range 8–10 ms post-stimulus) coincides with typical times for a fast EPSP observed in an intracellular recording after pathway stimulation, and the “fast hyperpolarization” (peak measurement range 28–68 ms post-stimulus) coincides in time with an intracellular inhibitory postsynaptic potential (IPSP). We labeled this component the “fast” hyperpolarization only to distinguish it in time from any hyperpolarization that might occur during the subsequent “slow component” time period (see below), and do not mean to imply any relative dependence on GABA_A_ versus GABA_B_ currents. The last interval (arbitrarily defined as 60–240 ms after the stimulus), we term simply the “slow component” as the signal during this epoch was most often a slow depolarization, although depending on experimental conditions it might be a depolarization, a hyperpolarization or both.

**Figure 4:**
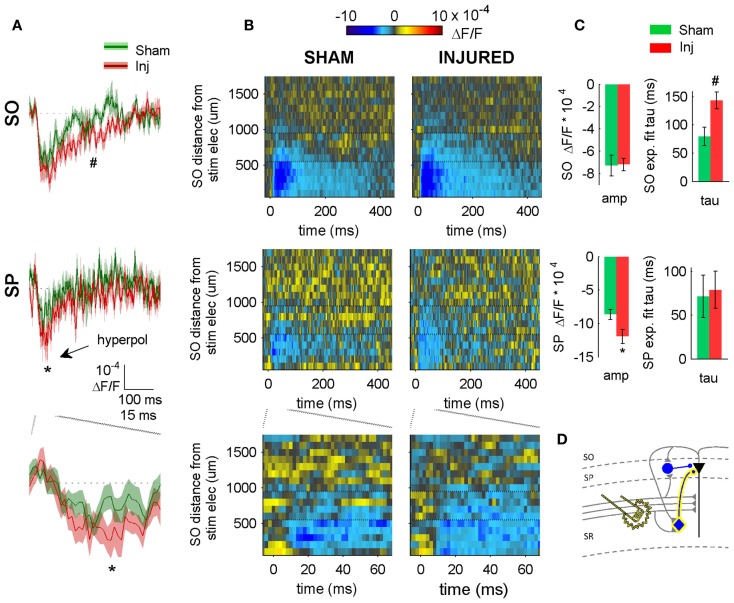
**SR-evoked hyperpolarization increase in SP and SO persists when excitatory synaptic transmission is blocked**. **(A)** Sham and injured group average VSD traces in SP and SO in response to SR-stimulation in solution containing APV and CNQX to block excitatory synaptic transmission. Note, SR-evoked hyperpolarization increases in SP and SO after injury. **(B)** Raster representation of VSD signal shows spatiotemporal extent of hyperpolarization increase. Persistence of hyperpolarization increase when excitatory synaptic transmission is blocked indicates that glutamatergic activation of interneurons does not contribute to the post-injury increase in hyperpolarization. Horizontal dotted lines indicate raster region corresponding to multi-segment analysis region (segments 6–9). **(C)** Group statistics indicating significant differences in hyperpolarization amplitude and duration (latter measured as exponential fit time constant over falling phase of response, beginning at 40 ms). **(D)** Schematic showing direct activation of CCK basket cell (yellow highlight around cell body and axon) by SR stimulation in APV and CNQX. Same legend as in Figure 1. Stimulus: 300 μA. Sham *n* = 11, injured *n* = 11. SO, stratum oriens; SP, stratum pyramidale; Inj, Injured; amp, amplitude.

**Figure 5:**
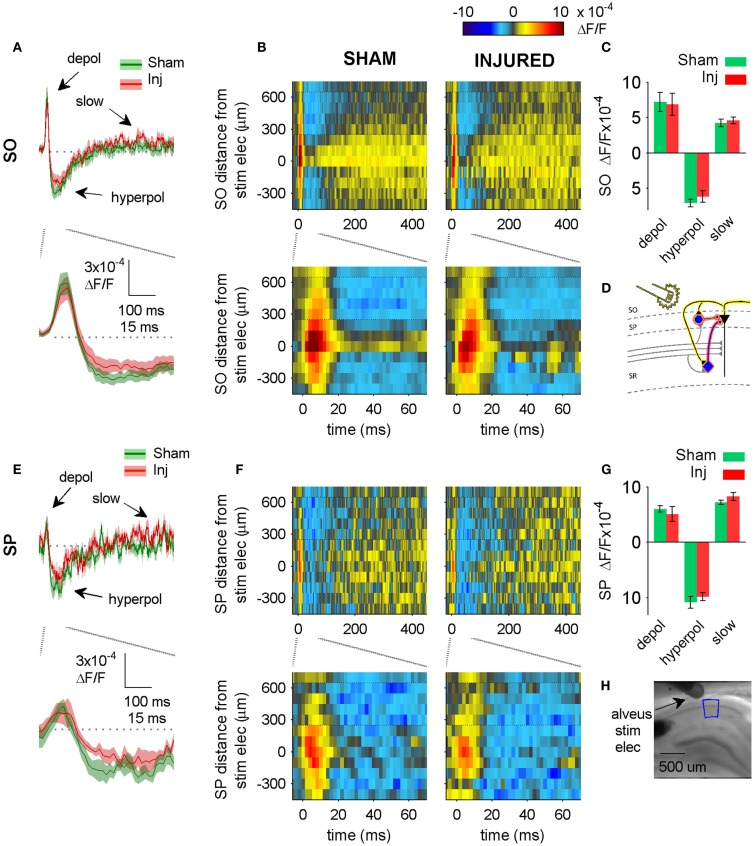
**Alveus-evoked responses in aCSF do not change after injury**. **(A,E)** Sham and injured group average VSD traces in SP and SO in response to alveus-stimulation in aCSF. The fast depolarization (0 – 15 ms) in the SO response to alveus stimulation is a combination of APs antidromically propagated toward CA1 pyramidal neuron somata, and the subsequent excitation of SO interneuron dendrites. No significant difference were observed in SP or SO depolarization (antidromic APs plus interneuron dendritic EPSPs) or hyperpolarization. The absence of any changes in depolarization suggests that feedback excitation of interneuron SO dendrites does not change after injury (see also Figure 6 in which the APV and CNQX insensitive component of the depolarization does not change after injury). **(B,F)** Sham and injured group average rasters for same slices as in **(A)**. Again, no significant differences were observed in any of the evoked responses. Positive distances are toward subiculum, negative distances are toward CA3. Horizontal dotted lines indicate raster region corresponding to multi-segment analysis region (segments 4–6, toward subiculum). **(C,G)** Group average statistics. No significant differences were observed. **(D)** Schematic showing antidromic activation of pyramidal neurons (yellow highlight), and direct (yellow highlight) plus synaptic (red highlight) activation of basket cells by alveus stimulation in aCSF. Schaffer collaterals are shaded gray to indicate non-activation by alveus stimulation. **(H)** Photomicrograph from a representative slice showing typical location of stimulating electrode and region used for analysis of alveus-stimulated responses. SO, stratum oriens; SP, stratum pyramidale; Inj, Injured; depol, depolarization; hyperpol, hyperpolarization; stim elec, stimulating electrode.

The character and spatial extent of the VSD responses varied according to the experimental conditions (Figures 1–5). The spatial location of the analysis region for a given test condition was set to span an intermediate range far enough from the stimulating electrode to avoid response saturation but close enough to the stimulating electrode so that significant differences would not be lost in the recording noise. For a given test condition the same analysis region was used for both sham and injured groups. Statistics were performed on the indicated multi-segment raster regions using the peak value and not the average over an interval containing the peak, as the average is more strongly affected by the time interval chosen. Peak measurements have an offset attributable to noise, which in our recordings was approximately 2 × 10^−4^ ΔF/F and −2 × 10^−4^ ΔF/F for measures of peak depolarization and hyperpolarization respectively. Accordingly, all measurements of positive going (depolarizing) peaks would be expected to have approximately 2 × 10^−4^ ΔF/F added to them due to noise, all measurements of negative going (hyperpolarizing) peaks would be expected to have approximately −2 × 10^−4^ ΔF/F added by noise.

**Figure 6:**
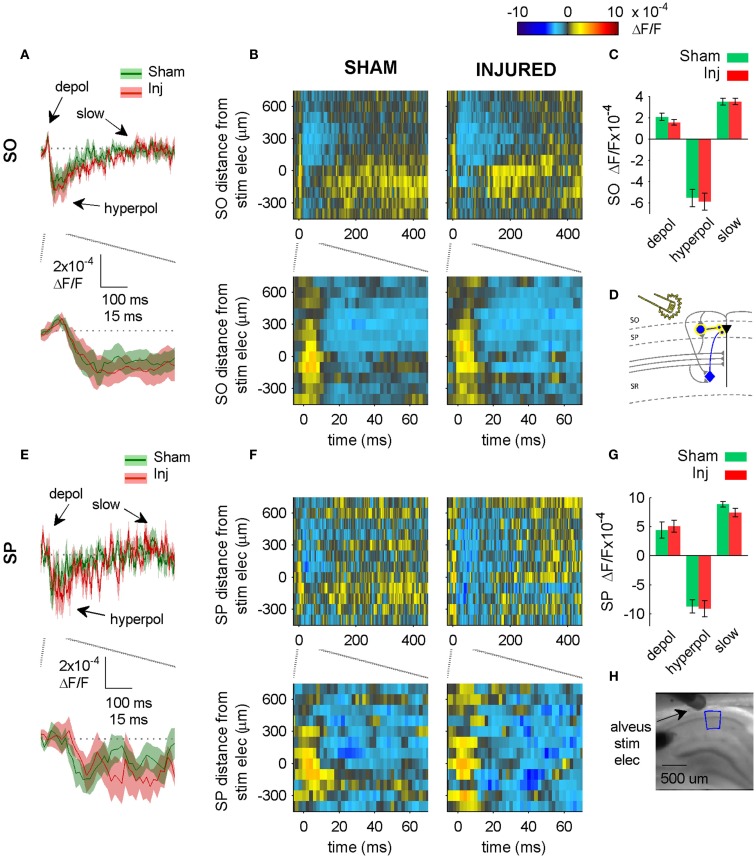
**Alveus-evoked responses in APV plus CNQX do not change after injury**. **(A,E)** Sham and injured group average VSD traces in SP and SO in response to Alveus-stimulation in APV (50 μM) and CNQX (6 μM) do not change after injury. Same slices as in Figure 4. With glutamatergic transmission blocked, responses are due to direct stimulation of interneurons. No significant differences were observed in SP or SO hyperpolarization, indicating no net functional changes at GABAergic synapses in SP or SO. **(B,F)** Sham and injured group average rasters for same slices as in **(A,E)**. Again, no significant differences were observed in SP or SO, indicating no net functional changes at GABAergic synapses in SP or SO. **(C,G)** Group average statistics. No significant differences were observed. **(D)** Schematic showing direct activation (yellow highlight) of interneuron synapses in SP and SO. Schaffer collaterals are shaded gray to indicate non-activation by alveus stimulation. Pyramidal neuron axons are shaded gray to emphasize that glutamatergic transmission has been blocked. Same legend as in Figure 1A. **(H)** Photomicrograph from a representative slice showing typical location of stimulating electrode and region used for analysis of alveus-stimulated responses. Positive distances from electrode are toward subiculum, negative distances are toward CA3. Stimulus: 100 μA. Sham *n* = 9, injured *n* = 8. Abbreviations: SO, stratum oriens; SP, stratum pyramidale; Inj, Injured; depol, depolarization; hyperpol, hyperpolarization; stim elec, stimulating electrode.

## Results

### Post-injury increase in SO hyperpolarization in response to SR stimulation

Previous reports demonstrated a decrease in SR-evoked and recorded CA1 field potentials 6–8 days post-injury (Witgen et al., 2005; Norris and Scheff, 2009; Cole et al., 2010), so we began by evaluating CA1 SR, SP, and SO VSD signals in response to SR stimulation to determine if VSD signals showed a similar post-injury decrease in SR-evoked responses. Figure 1 shows the experimental set-up and VSD movie frames recorded from representative slices from sham and injured animals (see also Videos S1 and S2 in Supplementary Material). Figure 2 shows regional average and raster representations of the group data for sham and injured mice. In slices from both the sham and injured animals the depolarization and subsequent hyperpolarizing drop have the right polarity and time course (Figures 1C and 2A–C) to be the VSD correlate of the EPSP/IPSP sequence typically observed in intracellular recordings (Shepherd, 2004; Ang et al., 2005). A post-injury decrease in SR-evoked SR-depolarization is visible in the representative slice movie frames as fewer red pixels near the stimulating electrode in SR, in the group average traces as a decrease in the initial upward deflection in SR (0–20 ms following the stimulus in Figure 2A, lower middle and bottom panels), and in the group rasters as fewer red pixels in SR (Figure 2B lower middle and bottom rows, raster sham peak 20.5 ± 1.6 × 10^−4^ ΔF/F, *n* = 25; injured peak 14.5 ± 1.8 × 10^−4^ ΔF/F, *n* = 22; *P* = 0.009). In simultaneous SR field potential recordings, the initial slope, which reflects bulk current flow in SR, also decreased significantly (Figure 2E, sham −0.334 ± 0.043 mV/ms, *n* = 23; injured −0.206 ± 0.036, *n* = 22; *P* = 0.025), in agreement with previous field potential reports (Witgen et al., 2005; Norris and Scheff, 2009; Cole et al., 2010). In summary, both the VSD recordings and the field potential recordings indicate a decrease in SR-evoked depolarizing responses.

Previous studies had also shown an increase in the amplitude of inhibitory postsynaptic currents during single-cell recordings at the cell body of pyramidal cells (Witgen et al., 2005). We observed a corresponding increase in hyperpolarization, but surprisingly, the increase did not occur near the stimulating electrode in SR, but rather in SO. This post-injury increase in SR-evoked SO hyperpolarization is visible in the representative movie frames as more blue pixels in SO (Figure 1C, hyperpol frames), and in the group average traces as a drop to lower levels in SO (25–100 ms following the stimulus, Figure 2A, upper panel; average trace sham peak −0.1 × 10^−4^ ΔF/F, injured peak −2.9 × 10^−4^ ΔF/F), and in the rasters as more dark blue pixels in SO and more hyperpolarized peak values (Figure 2B upper panels; sham peak hyperpolarization −4.4 ± 0.6 × 10^−4^ ΔF/F, *n* = 25; injured −6.2 ± 0.7 × 10^−4^ ΔF/F, *n* = 22; *P* = 0.042). The spatial distribution of the evoked responses in SO also changed after injury, with hyperpolarization appearing closer to the stimulating electrode (Figure 2B, upper panel, increased number of blue pixels over the range from 300 to 500 μm. See also Figure 3). Following the trough of the fast hyperpolarization, the VSD signal typically rose over the next 50–150 ms, and then slowly returned to pre-stimulus levels. This slow portion of the VSD response has not been well studied, and may have a glial component (Konnerth et al., 1987; Kojima et al., 1999). In SR the peak of the slow polarization was smaller but not significantly different (Figures 2A–C, sham 10.5 ± 0.8 × 10^−4^ ΔF/F, *n* = 25; injured 8.1 ± 0.8 × 10^−4^ ΔF/F, *n* = 22, *P* = 0.054). In SO the slow polarization was significantly smaller (Figures 2A–C, sham 5.4 ± 0.5 × 10^−4^ ΔF/F, *n* = 25; injured 3.7 ± 0.5 × 10^−4^ ΔF/F, *n* = 22, *P* = 0.026). Overall, the SR-stimulation results confirm the CA1 inhibition increase previously reported in single-cell studies (Witgen et al., 2005), and extend those findings to reveal that the site of expression of the increased inhibition is limited largely to SO and adjacent SP. Knowing where the post-injury increase in hyperpolarization occurs can provide insight regarding the type of cells responsible for the increase.

### Source of SR-evoked increase in SO hyperpolarization

Inhibition observed following pathway stimulation is a mixture of feedback, feedforward, and directly activated inhibition. To determine the extent to which the SR-evoked SO hyperpolarization increase was dependent on the glutamatergic excitation of interneurons, we repeated the SR stimulation in solution containing APV (50 μM) and CNQX (6 μM) to block glutamatergic transmission. In APV and CNQX, the evoked response is due primarily to (1) directly evoking APs in interneuron somata and/or axons near the stimulating electrode (2) propagation of these directly evoked interneuron APs to interneuron efferent synapses, and (3) GABAergic currents produced by those interneuron synapses. The SR-evoked SO and SP response in APV and CNQX was entirely hyperpolarizing (Figure 4), and a fast hyperpolarization difference persisted between slices from sham and injured animals in SP as an increase in amplitude, and in SO as an increase in duration (amplitudes: sham SP −8.7 ± 0.7 × 10^−4^ ΔF/F, *n* = 11; injured SP −11.9 ± 1.1 × 10^−4^ ΔF/F, *n* = 11; *P* = 0.022; sham SO −7.3 ± 0.9 × 10^−4^ ΔF/F, *n* = 11, injured SO −7.2 ± 0.6 × 10^−4^ ΔF/F, *n* = 11, *P* = 0.896; decay phase single exponential fit time constants (tau): sham SP 72 ± 24 ms, *n* = 7; injured SP 79 ± 21 ms, *n* = 9, *P* = 0.758; sham SO 79 ± 16 ms, *n* = 9, injured SO 143 ± 15 ms, *n* = 9, *P* = 0.019). The persistence of an SR-evoked SO hyperpolarization increase during glutamatergic transmission blockade, indicates that the post-injury increase in SR-evoked SO hyperpolarization was primarily due to the direct electrical activation of interneurons.

We tested for net functional changes at inhibitory synapses in SO by washing in solution containing APV and CNQX to block excitatory synapses, and then stimulating the alveus (near the alveus-SO border) to directly activate interneuron axons and synapses in SO (Figure 5). No significant differences were observed in the alveus-evoked responses in SP or SO in either control aCSF (Figure 5) or in solution containing APV and CNQX (Figure 6) suggesting that there were no net functional changes at GABAergic synapses in SP or SO, and that the increase in SR-evoked SO hyperpolarization was not due to a net increase in SP or SO inhibitory synaptic function. Since we observed an increase in SR-evoked SO hyperpolarization, but did not observe an increase in SO inhibitory neuron synaptic strength, this suggests that the increase in SR-evoked SO hyperpolarization was due to an increase in the production and/or propagation of APs in interneurons that have somata and/or axons in SR (near our SR stimulating electrode), and efferent synapses in SP and SO.

**Figure 7:**
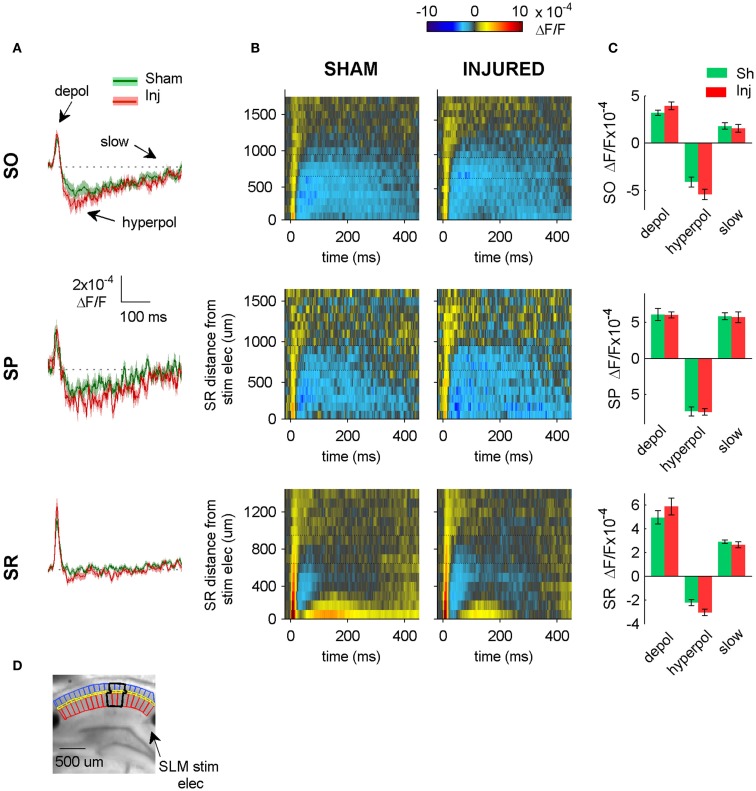
**SLM-evoked responses in aCSF do not change after injury**. **(A)** Group average regional VSD signals in SO (top panel), SP (middle panel), and SR (bottom panel) in response to SLM stimulation in aCSF. SLM-evoked hyperpolarization does not change significantly after injury (SO, *P* = 0.114; SP, *P* = 0.926; SR, *P* = 0.059 sham *n* = 13, injured *n* = 11). The absence of any significant change in the SR, SP, or SO responses to SLM stimulation suggests that inadvertent stimulation of SLM does not contribute significantly to SR-evoked SO hyperpolarization. **(B)** Raster plots also show no change in SP or SO hyperpolarization. Horizontal dotted lines on rasters indicate analysis region. **(C)** Group data shows no significant differences in the SO responses to SLM stimulation, although there was a moderate increase in SR hyperpolarization. **(D)** Photograph of representative slice depicting analysis region (black outline) and location of SLM stimulating electrode. Stimulus: 300 μA. Sham *n* = 13, injured *n* = 11. SO, stratum oriens; SR, stratum radiatum; SLM, stratum lacunosum-moleculare; depol, depolarization; hyperpol, hyperpolarization; stim elec, stimulating electrode.

To test for possible inadvertent co-stimulation of SLM, which might have contributed to the SO hyperpolarization increase during the SR-stimulation experiments, and to evaluate SLM signaling after injury, we stimulated SLM directly. We did not observe any significant differences in SP or SO when SLM was stimulated (Figure 7), suggesting that the SR-evoked SO hyperpolarization increase was not affected by inadvertent SLM co-stimulation, and was in fact due solely to SR stimulation.

### Reduction in evoked action potentials after injury

**Figure 8:**
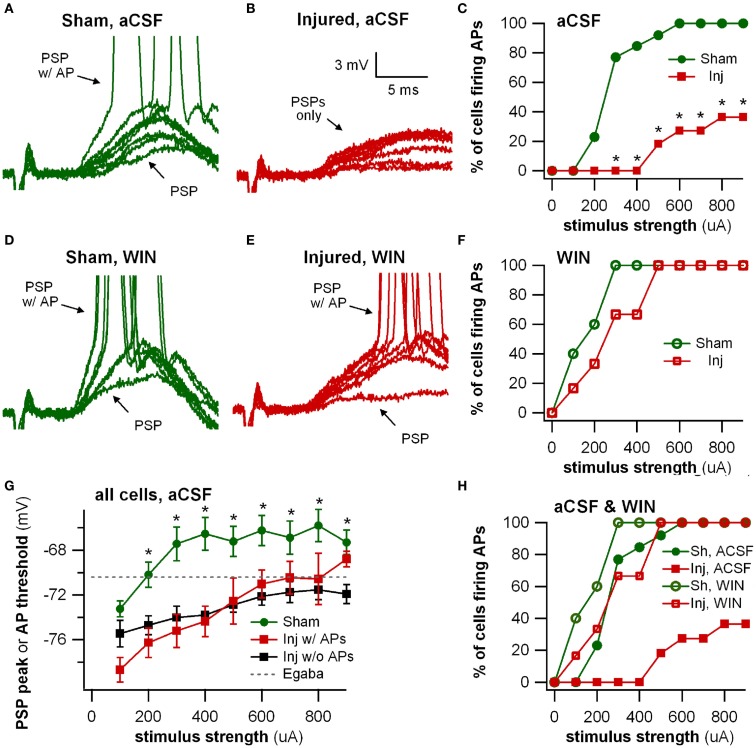
**Stimulus-evoked action potentials (APs) are reduced after injury, and restored to normal when GABAergic signaling by CCK positive interneurons is suppressed**. Whole-cell current clamp recordings from individual CA1 pyramidal neurons in response to SR stimulation in aCSF and in solution containing the cannabinoid agonist WIN55,212-2. **(A)** Representative whole-cell current clamp recording in aCSF in slice prepared from sham animal. The postsynaptic response and AP probability increase with increasing stimulus strength. **(B)** In a majority of the whole-cell recordings in slices prepared injured animals, APs could not be evoked in control aCSF even at the strongest stimulus strength, and the average postsynaptic response reached a plateau level which responses did not exceed, even as stimulus strength was increased (see also G below). **(C)** Percent of cells firing APs in aCSF versus stimulus strength. After injury, the percent of cells firing APs is significantly lower. **(D–F)** Whole-cell current clamp recordings from the same cells as in **(A–C)**, after treatment with the CB1 agonist WIN55,212-2 (100 nM), which selectively suppresses GABA release from CCK basket cells. WIN55,212-2 increases AP firing in cells from slices prepared from injured animals to a level not significantly different from either sham cells treated with WIN55,212-2 or sham cells in aCSF. **(G)** PSP peak or AP threshold (defined as AP threshold during trials which evoked APs, and as PSP peak voltage during trials which did not produce APs) versus stimulus strength in aCSF. The average initial response of cells from sham animals rises well above the reversal potential for GABA_A_ and approaches the average EPSP-triggered AP threshold (−66.2 ± 1.3 mV, *n* = 13) for stimuli of 300 μA and larger. After injury the average response in cells which did not fire APs reached a ceiling level which it did not exceed even as the stimulus strength was increased. This peak voltage was close to the reversal potential for GABA_A_ IPSPs. Asterisks indicate significant differences between sham and injured without APs (Mann–Whitney *U*-test, Bonferroni corrected, *P* < 0.025). **(H)** Superimposition of AP percent versus stimulus strength for aCSF **(C)** and WIN55,212-2 **(F)**. WIN55,212-2 restores injured cell AP firing to levels not significantly different from sham. Trials containing APs were truncated after initial portion. Stimulus artifact partially blanked. Recording baselines were adjusted to the average pre-stimulus membrane potential. Stimuli: 100–900 μA in 100 μA increments. APs, action potentials; PSP, postsynaptic potential; Sh, Sham; Inj, Injured.

Having observed a post-injury increase in SR-evoked SP and SO hyperpolarization, we hypothesized that this augmented hyperpolarization would be associated with a decrease in stimulus-evoked APs in CA1 pyramidal neurons. We tested for a decrease in CA1 stimulus-evoked APs by recording AP firing in response to SR stimulation, during whole-cell current clamp recordings from individual CA1 pyramidal neurons. Stimuli ranging from 100 to 900 μA in 100 μA increments were delivered to slices from both sham and injured animals. APs were triggered by the stimulus-evoked EPSP in 100% of the cells in slices from sham mice but only 36.4% of the cells in slices from injured mice (Figures 8A–C; *n* = 13 sham, *n* = 11 injured, *P* = 0.001, Fisher exact test), despite the inclusion of comparatively large stimuli (e.g., stimuli well above the level which produced large, saturating responses in the field potential recordings). For the minority of cells in slices from brain injured mice in which the EPSP did evoke an AP, the minimum stimulus current required to generate an AP was significantly higher (sham 323 ± 31 μA, *n* = 13; injured 600 ± 61 μA, *n* = 4; *P* = 0.008), although there were no differences between injured and sham in intrinsic AP threshold (measured by current injection through the whole-cell recording electrode), evoked AP threshold (voltage at which an AP was triggered during an evoked EPSP), input resistance, or resting membrane potential (Table 1). The post-injury decrease in the number of SR-evoked APs in the pyramidal neuron recordings is consistent with the increase in SR-evoked inhibition in the VSD recordings.

**Table 1:** **Intrinsic membrane properties and excitability of CA1 pyramidal neurons**.

	aCSF		WIN		
	Sham	Injured	Sham	Injured	
Membrane potential (mV)	−67.5 ± 1.4 (13)	−69.6 ± 1.5 (13)	n/a	n/a	
Input resistance (MΩ)	182 ± 10 (12)	185 ± 12 (13)	157 ± 48 (4)	146 ± 14, (6)	
Intrinsic AP threshold (mV)	−60.5 ± 1.4 (12)	−61.1 ± 1.5 (13)	−66.2 ± 1.8 (4)	−65.4 ± 1.9 (6)	
Evoked AP threshold (mV)	−66.2 ± 1.3 (13)	−70.5 ± 1.3 (4)	−68.2 ± 1.1 (5)	−67.5 ± 1.7 (6)	
Min. stim. to evoke AP (MΩ)	323 ± 31 (13)	600 ± 61 (4)	200 ± 40 (5)	317 ± 60 (6)	

	**Sham aCSF vs. injured aCSF**	**Sham aCSF vs. Sham WIN**	**Sham aCSF vs. injured WIN**	**Injured aCSF vs. injured WIN**	**Sham WIN vs. injured WIN**

Kruskal–wallis *P*-values for indicated comparisons
Input resistance	>0.99	0.668	0.368	0.422	>0.99
Intrinsic AP threshold	0.99	0.282	0.355	0.796	>0.99
Evoked AP threshold	0.562	>0.99	>0.99	>0.99	>0.99
Min. stimulus to evoke AP		0.0498	0.329		0.075

*Mann–Whitney *U*-test *P*-value for resting potential: *P* = 0.361*.

**Figure 9:**
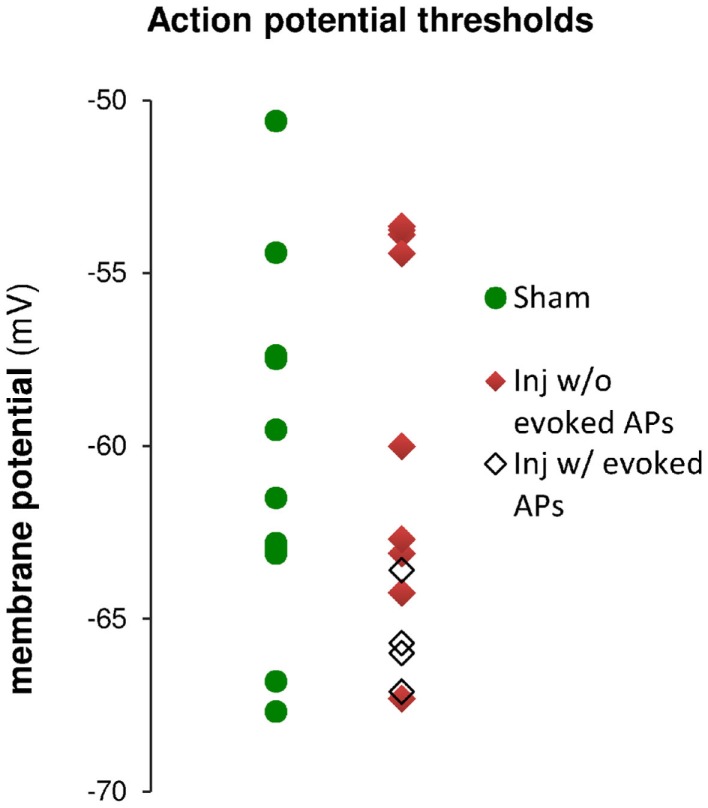
**Action potential (AP) thresholds measured by current injection through the whole-cell recording electrode during current clamp recordings in cells from sham and injured animals**. The range of action potential thresholds overlapped, and the mean values were not different between cells from sham and injured animals (see also Table 1), indicating no change in pyramidal neuron AP threshold after injury. Note that the injured cells in which stimulus-evoked action potentials could be triggered off the EPSP were those cells for which the AP threshold was near the low end of the AP threshold range, closer to E_GABA_A__ (−70.4 mV).

*In control, aCSF no significant differences were observed between cells from sham and injured animals in membrane potential, input resistance, AP threshold, or the voltage during an EPSP at which an AP was triggered. WIN55,212-2 also had no effect on any of these parameters (i.e., sham aCSF parameters were not significantly different from WIN aCSF parameters). The absence of any injury-induced changes in intrinsic parameters (membrane potential, input resistance, intrinsic, and evoked AP thresholds) indicates that the decreased AP output after injury is not due to changes in intrinsic parameters, and the absence of any WIN55,212-2 effect on these parameters indicates that the restoration of AP firing was also not due to any WIN55,212-2 effect on intrinsic parameters. There was a significant increase in the stimulus current required to evoke an action potential after injury, however, and this difference was reduced by application of 100 nM WIN55,212-2. Note that for injured cells the stimulus-evoked AP threshold can only be measured from the minority of injured cells which responded to the stimulus by firing APs, and that such sampling is likely to be skewed toward including cells with more hyperpolarized AP thresholds (e.g., responder intrinsic AP threshold −65.6 ± 0.7 mV, *n* = 4, non-responder intrinsic AP threshold −58.0 ± 2.0 mV, *n* = 7, Mann–Whitney *U*-test *P* = 0.073; see also Figure 9). The more hyperpolarized thresholds for stimulus-evoked APs compared to intrinsic APs generated by current injection at the cell body may reflect differences in where the AP is initiated by the two different protocols (Stuart et al., 1997). Values listed are mean ± SE. Number of recordings listed in parentheses*.

*WIN, WIN55,212-2*.

To further explore the possibility that an increase in inhibition was responsible for the decrease in stimulus-evoked APs, we examined the peak postsynaptic membrane voltage during a stimulus-evoked PSP (postsynaptic potential). When strong GABA_A_ inhibitory synaptic currents are activated in a neuron, they tend to drive the membrane voltage toward the GABA_A_ reversal potential (E_GABA_A__), a phenomenon known as “shunting” when E_GABA_A__ is close to the resting membrane potential. If E_GABA_A__ is lower (more hyperpolarized) than the AP threshold, as it is in our solutions, such shunting can prevent the postsynaptic neuron from reaching its AP threshold. We can test whether membrane potential shunting by GABAergic inhibitory neurons was contributing to the reduction in AP firing after injury by comparing E_GABA_A__ to the peak postsynaptic membrane voltage after the stimulus. For responses that did not generate APs, the peak PSP voltage was used; for responses that did generate APs, the peak voltage was defined as the AP threshold. In injured cells that did not fire stimulus-evoked APs, the average peak voltage reached a plateau level well below the AP threshold, and did not rise above this sub-threshold level even as the stimulus strength was increased. In slices prepared from injured animals, the cells that did not fire stimulus-evoked APs had a maximum peak voltage of −70.0 ± 1.2 mV (*n* = 7), which is very close to the estimated E_GABA_A__ of −70.4 mV for our solutions, but well below the intrinsic AP threshold for those non-firing cells (−63.5 ± 1.6 mV, *n* = 9). The injured cells, which fired stimulus-evoked APs were those cells for which the intrinsic AP thresholds were at the low end of the range (Figure 9), and close enough to E_GABA_A__ for the action potential onset currents to overcome inhibitory membrane shunting. In summary, for the injured cells, which did not fire APs, the similarity between the peak response voltage and E_GABA_A__ supports the hypothesis that an increase in GABA_A_ conductance was shunting responses after injury, thus diminishing the ability of CA1 pyramidal neurons to fire APs.

### WIN55,212-2 sensitive interneurons contribute to reduction in evoked action potentials after injury

**Figure 10:**
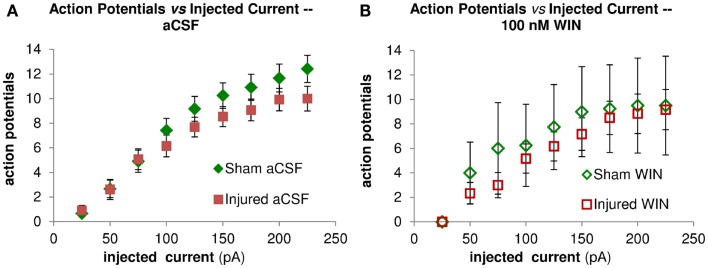
**Repetitive action potential (AP) firing is not altered by injury and is unaffected by treatment with the cannabinoid agonist WIN55,212-2**. CA1 Pyramidal neuron voltage response to current injection through recording electrode. **(A)** Average AP frequency in response to depolarizing current steps (0–225 pA, in 25 pA increments) in aCSF. No significant differences in AP frequency were found between sham (*n* = 13) and injured (*n* = 13) pyramidal neurons at any of the current steps. **(B)** Average AP frequency in response to depolarizing current steps (0–225 pA, in 25 pA increments) in aCSF containing 100 nM WIN55,212-2. No significant differences in AP frequency were found between sham (*n* = 4) and injured (*n* = 6) pyramidal neurons at any of the current steps; nor did WIN55,212-2 significantly affect AP frequency in either condition. These results are consistent with a lack of any changes in intrinsic membrane properties (see also Figure 9 and Table 1).

**Figure 11:**
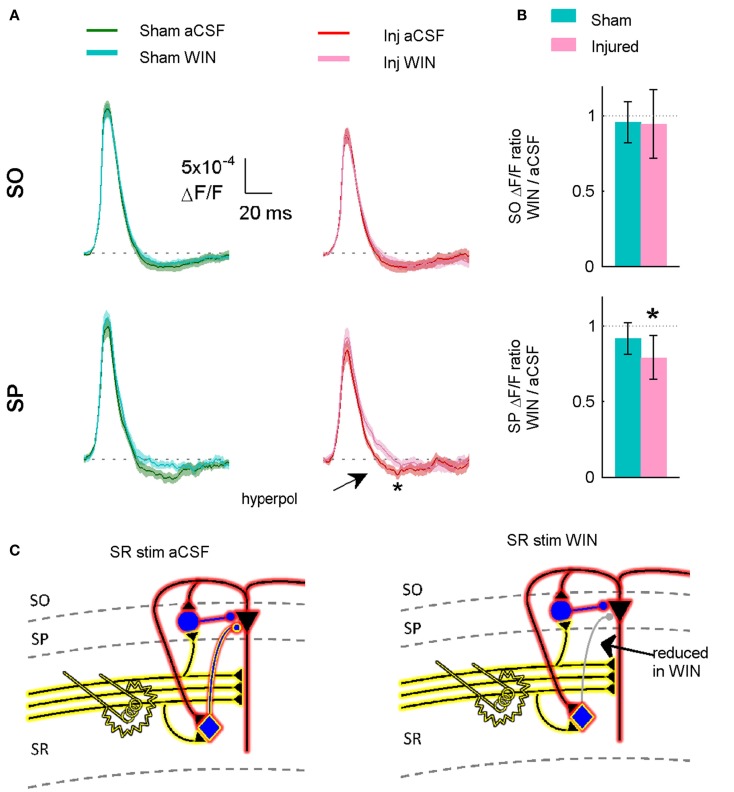
**Suppressing GABAergic signaling by CCK positive interneurons significantly reduces hyperpolarization in slices prepared from injured animals**. **(A)** VSD average traces for slices prepared from sham and injured animals in control aCSF and in the cannabinoid agonist WIN55,212-2 (*n* = 12 for both groups). In slice prepared from injured mice, the same concentration (100 nM) of WIN55,212-2 that significantly increased action potential firing in the pyramidal neuron recordings, significantly reduced SP hyperpolarization in VSD recordings, indicating that cannabinoid-sensitive interneurons contribute to both phenomena. Although WIN55,212-2 reduced SR-evoked SP hyperpolarization in both sham and injured slices, the difference was only significant for injured animals (sham *P* = 0.226, injured *P* = 0.032). Stimulus 500 μA, analysis region 1100–1400 μm. **(B)** Group average WIN55,212-2 effect. Ratio of response in WIN55212-2 to response in aCSF. The relative magnitude of the WIN effect is significantly different from unity in the slices from injured animals (signed rank *P* values for test of median = 1: sham *P* = 0.424, injured *P* = 0.042). Dotted line indicates WIN/aCSF ratio equal to unity. **(C)** Schematic showing reduction of synaptic transmission from CCK *positive* basket cell to pyramidal neuron by WIN55,212-2.

Action potential firing in pyramidal neurons is modulated by perisomatic inhibition (Miles et al., 1996), which is provided primarily by basket cell interneurons (Freund and Katona, 2007). Basket cells can be divided into two mutually exclusive groups by the presence of PV or CCK, respectively (Klausberger et al., 2005). Both types of basket cells project to the SP and adjacent portions of SO and SR (Glickfeld and Scanziani, 2006; Foldy et al., 2010) although the comparatively sparse projection into SR is stronger for CCK basket cells (Glickfeld and Scanziani, 2006). The somata of CCK basket cells, but not PV basket cells, can also be found in SR (Freund and Buzsaki, 1996; Lee et al., 2010), which may make CCK basket cells more likely to be activated by a stimulating electrode in SR. APs evoked in the SR somata or axonal branches of basket cells and then propagating through the extensive basket cell arbors would produce responses consistent with our VSD data as well as the decreased AP firing in the pyramidal neuron recordings. GABA release from CCK basket cells (but not PV basket cells) is remarkably sensitive to modulation by presynaptic cannabinoid type 1 (CB1) receptors (Glickfeld et al., 2008; Lee et al., 2010), so we tested the hypothesis that increased GABAergic signaling by cannabinoid-sensitive interneurons was responsible for the post-injury reduction in AP firing by adding a low concentration (100 nM) of the CB1 agonist WIN55,212-2 to the bath, and repeating the SR stimulation. WIN55,212-2 restored AP firing in cells from injured animals to a level not significantly different from sham (Figures 8D–F,H). In addition, all of the pyramidal neurons, which previously failed to fire APs (7 out of 11), now fired APs (11 out of 11). WIN55,212-2 did not change input resistance, AP threshold or the voltage during an EPSP at which APs were triggered (Table 1), or the AP frequency versus current injection (Figure 10). WIN55,212-2 also reduced hyperpolarization in the VSD recordings of slices prepared from injured animals (Figure 11). The WIN55,212-2 results above support the hypothesis that an increase in inhibition from CCK positive interneurons contributes to the decrease in stimulus-evoked APs after injury.

To determine whether or not 100 nM WIN55,212-2 was acting at excitatory terminals (see also Discussion), we measured the initial stimulus-evoked excitatory response in pyramidal neurons by integrating those responses over the first 2 ms of the response, an interval during which any polysynaptic inhibitory contribution to the evoked response would likely be minimal (Glickfeld and Scanziani, 2006). WIN55,212-2 did not have a significant effect on the initial excitatory responses in slices from either sham or injured animals (Figure 13), indicating that it was acting on GABAergic transmission, and not glutamatergic transmission. The initial slope of SR-evoked SR field potentials is another measure of excitatory synaptic transmission, and these were also unaffected by WIN55,212-2, in slices from both sham and injured mice (Figure 13). Regarding the possibility that injury may have altered endocannabinoid signaling at excitatory synapses we note that the SR-evoked and recorded field potential paired pulse ratio did not change after injury (sham 1.55 ± 0.07, *n* = 12; injured 1.57 ± 0.03, *n* = 9; *P* = 0.972; 75 ms inter-stimulus interval, 300 μA stimulus), while presynaptic CB1-mediated changes in glutamatergic responses typically are associated with an increased paired pulse ratio (Ameri et al., 1999; Ohno-Shosaku et al., 2002; Kawamura et al., 2006; Hoffman et al., 2007; Glickfeld et al., 2008). Taken together, these results suggest that WIN55,212-2 was acting primarily at GABAergic and not glutamatergic synapses, and support the hypothesis that an increase in GABAergic signaling by WIN-sensitive interneurons contributes to the decrease in CA1 output after injury.

**Figure 12:**
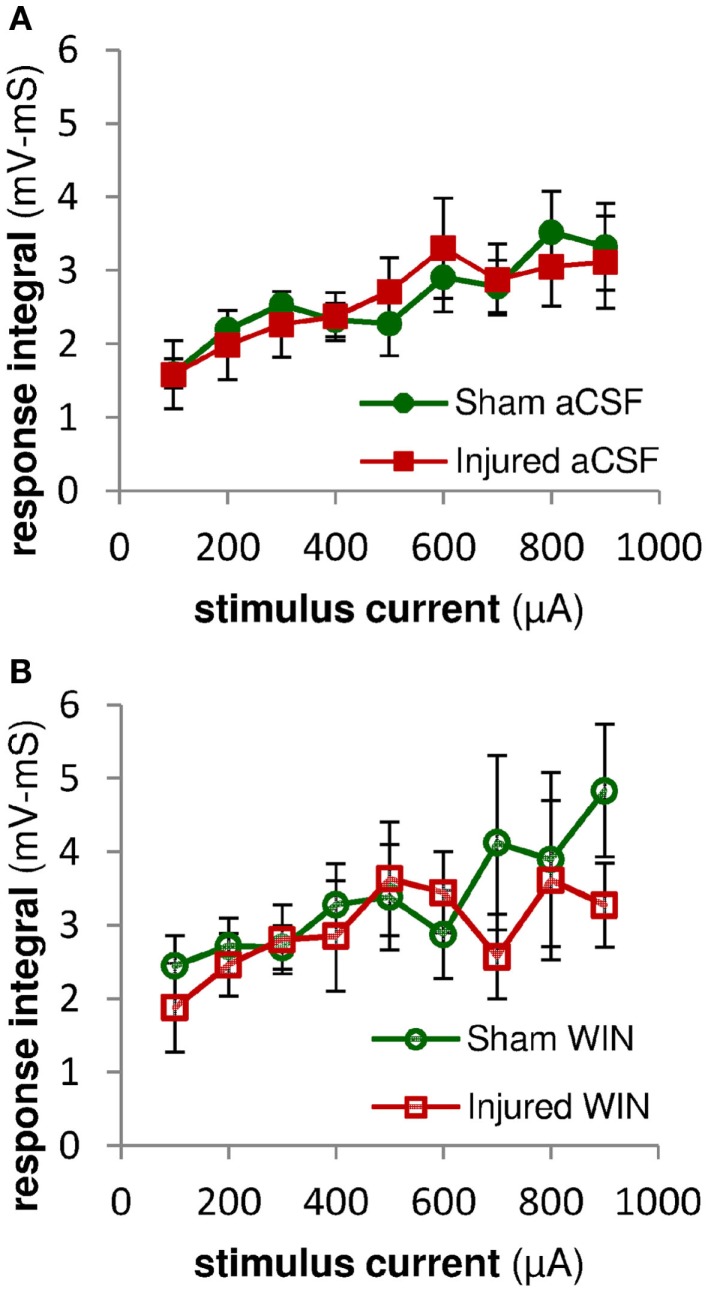
**Excitatory synaptic responses in pyramidal neuron recordings were not altered by injury or treatment with the cannabinoid agonist WIN55,2122**. Integral of SR-evoked responses in whole-cell current clamp recordings from CA1 pyramidal neurons over the first 2 ms after the response onset. Since action potentials began as early as 2 ms after the onset of the stimulus-evoked responses, we assessed the excitatory synaptic component of the evoked response by integrating the response over the first 2 ms after the response onset. During this early time period, stimulus-evoked synaptic inhibition would not yet be active, and so the integral is a measure of excitatory synaptic activity (action potentials can be distinguished from synaptic potentials by the rate of rise of the response – see Sections “Materials and Methods” and “Analysis”). **(A)** No significant differences were observed between the excitatory responses in pyramidal neurons from sham versus injured mice, suggesting that injury did not alter the excitatory component of the evoked synaptic responses. **(B)** Integral over first 2 ms of stimulus-evoked pyramidal neuron response as in **(A)** above. Treatment with WIN55,212-2 (100 nM) did not significantly affect excitatory synaptic responses in either sham or injured animals, nor the difference between sham and injured. Mann-Whitney paired comparisons for 100–900 μA stimuli (minimum, maximum, and median *P* values): sham aCSF versus sham WIN, 0.151, 0.999, 0.421; injured aCSF versus injured WIN, 0.394, 0.999, 0.931; sham aCSF versus injured aCSF 0.537, 0.999, 0.841; sham WIN versus injured WIN, 0.330, 0.931, 0.662. Kruskal–Wallis comparison across all groups for 100–900 μA stimuli: 0.619, 0.653, 0.796, 0.685, 0.529, 0.913, 0.806, 0.954, 0.555.

**Figure 13:**
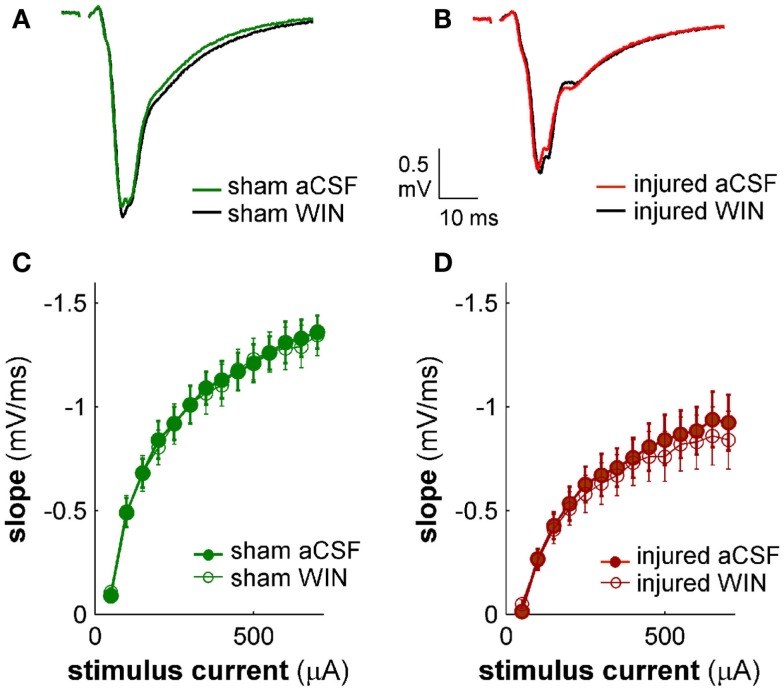
**Cannabinoid agonist WIN55,212-2 does not affect excitatory transmission in CA1 field potential recordings**. CA1 SR-evoked and recorded field potentials. **(A,B)** Ten-trial average SR-evoked SR field potentials for representative sham **(A)** and injured **(B)** mouse brain slices in normal aCSF and in aCSF containing 100 nM WIN55,212-2. Stimulus 300 μA. WIN55,212-2 did not have a significant effect on the initial portion of the field potentials in either sham or injured mouse brain slices, indicating that WIN55,212-2 did not significantly affect SR-evoked excitatory responses in SR. **(C,D)** Group average field potential slope versus stimulus current for slices from sham **(C)** and injured **(D)** mice. The slope in the early portion of an SR field potential is a measure of excitatory synaptic transmission. WIN55,212-2 did not significantly affect either sham or injured field potential slopes at any stimulus strength tested, again indicating that WIN did not significantly affect SR-evoked excitatory synaptic transmission. Wilcoxon Signed Rank *P* values >0.05 for all stimulus strengths. Sham *n* = 8, injured *n* = 8; data plotted as mean ± SE.

## Discussion

We discovered layer-specific changes in CA1 evoked responses after lFPI, including an increase in SR-evoked SO hyperpolarization, and a decrease in CA1 output, as measured by stimulus-evoked APs. Previous studies showed a decrease in CA1 excitability 6–8 days post-injury (Witgen et al., 2005; Schwarzbach et al., 2006; Norris and Scheff, 2009; Cole et al., 2010). Our results are consistent with these reports, yet provide layer-specific information regarding these changes and also identify a specific population of cells altered by injury.

An SR-evoked SO hyperpolarization increase persisted in the presence of APV and CNQX indicating that it was due primarily to the direct electrical activation of interneurons. In addition, the absence of any difference between sham and injured responses to direct stimulation of the alveus in solution containing APV and CNQX, indicates that there were no net functional changes at inhibitory synapses in SO. Taken together, these results suggest that the increase in SR-evoked SO hyperpolarization was due to an increased ability to evoke and/or propagate APs in interneurons with cell bodies and/or axons in SR and efferent synapses in SO. Consistent with these results, Ross and Soltesz (2000) reported interneuron depolarization in the dentate gyrus after lFPI and a lower stimulus threshold for evoking APs, although at 4 days this effect was no longer significant in the dentate gyrus.

Basket cells are the primary source of perisomatic inhibition, and also modulate AP initiation (Miles et al., 1996; Freund and Katona, 2007), making them good candidates for mediating both the increase in SR-evoked SO inhibition in the VSD recordings, and also the decrease in AP firing in the single-cell recordings. Supporting this, the single-cell recordings showed an increase after injury in the stimulus required to evoke an AP, and a decrease in the probability of evoking an AP. Injured cells that did not fire an AP had a peak postsynaptic response very near the reversal potential for GABA_A_, consistent with the hypothesis that an increased GABA_A_ current was mediating the decrease in AP firing after injury. This reduction in AP firing was eliminated by bath application of a low concentration (100 nM) of the CB1 receptor agonist WIN55,212-2, which selectively reduces GABA release from CCK basket cells (Lee et al., 2010). Although AP firing in WIN55,212-2 for cells in slices from injured mice was not significantly different from AP firing in aCSF for cells in slices from sham mice, WIN55,212-2 also caused a moderate increase in AP firing in sham cells, especially at lower stimulus strengths. It is possible, therefore, that WIN55,212-2 restored injured cell AP firing to normal aCSF sham levels by over-suppressing GABA release in slices from injured animals, compensating for a possible diminished excitatory responses in injured animals. Nonetheless, the WIN55,212-2 results are consistent with the hypothesis of an increase after injury in GABAergic signaling by cannabinoid-sensitive interneurons.

The current work takes a first step toward identifying the circuit-level mechanisms responsible for the decrease in CA1 network excitability following TBI. Although our data support the hypothesis of a post-injury increase in GABAergic signaling by cannabinoid-sensitive interneurons, determining whether or not this group includes the CCK positive basket cells will require a future study with technically demanding paired recordings between *post hoc* identified CCK basket cells and pyramidal neurons. Optogenetic or chemogenetic methods (e.g., Taniguchi et al., 2011) are not well-suited here; as there is no currently known combination of molecular markers, which uniquely targets CCK positive basket cells (Ascoli et al., 2008). That having been said, several lines of evidence make the CCK basket cells likely candidates. Our VSD results indicate that the affected interneurons had an axonal projection limited to SP and SO as we did not observe any injury-induced increases in hyperpolarization in SR. The only interneurons which project primarily to SP and SO, and not also to SR, are the PV basket cells, the CCK basket cells, and the axo-axonic (or chandelier) cells (reviewed in Freund and Katona, 2007; Klausberger and Somogyi, 2008). Not surprisingly, AP firing is also controlled primarily by these same three cell types (Miles et al., 1996; Freund and Katona, 2007). AP firing was robustly depressed after injury and restored to normal by a low concentration (100 nM) of the CB1 receptor agonist WIN. The effect on AP firing was dramatic (Figure 8), and unlikely to have been mediated by interneurons playing only a minor role in the regulation of AP firing. Of these three cell types, CB1 receptors are found only on CCK basket cells and are not found on either PV basket cells (Katona et al., 1999; Marsicano and Lutz, 1999) (functional assays in Glickfeld and Scanziani, 2006; Glickfeld et al., 2008) or axo-axonic interneurons (Takacs et al., 2014). While unequivocally proving the involvement of CCK basket cells will require paired recordings between CCK basket cells and pyramidal neurons, we note nonetheless that CCK basket cells are the only interneurons, which project primarily to SP and SO, are critically involved in regulating AP firing, and are sensitive to cannabinoids.

The presynaptic terminals of pyramidal neurons also contain CB1 receptors (Kawamura et al., 2006), although at much lower levels than interneurons (Katona et al., 2006), and their activation requires stronger stimulation (Ohno-Shosaku et al., 2002). In addition, there are considerable species and strain differences in the sensitivity of glutamatergic responses to WIN55,212-2 (Hoffman et al., 2005, 2010; Haller et al., 2007). Of particular relevance to the current study, Hoffman et al. (2005) recorded simultaneously in the same recording chamber and solutions from slices prepared from C57BL/6J mice (the strain used here) and Sprague Dawley rats, and found WIN sensitivity in the glutamatergic responses of Sprague Dawley rats (and also CD1 mice), but not C57BL/6J mice. In a follow-up study, Hoffman et al. (2010) found that C57BL/6J glutamatergic responses became WIN-sensitive only when activation of the A1 adenosine receptor was reduced, and noted that C57BL/6J mice have unusually high levels of endogenous adenosine and that adenosine signaling is modulated by a wide variety of factors. A1 receptors are not present on inhibitory neuron terminals, and the same authors did find that WIN55,212-2 reduced C57BL/6J GABAergic responses under control conditions (Hoffman et al., 2005). By contrast, Kawamura et al. (2006) and Takahashi and Castillo (2006) reported that WIN55,212-2 reduced glutamatergic responses, although care must be taken when interpreting the results of studies using WIN55,212-2 in the micromolar range, as such comparatively high concentrations have been shown to have non-specific effects (Shen and Thayer, 1998; Oz, 2006; Pertwee, 2010). There may also be other factors underpinning the variability of glutamatergic responses to WIN55,212-2 (reviewed in Ohno-Shosaku et al., 2002).

In normal, aCSF the SR-evoked hyperpolarization increase was significant in SO but not in SP. In APV and CNQX significant effects were observed in both layers. In WIN55,212-2 significant effects were seen in SP but not SO. As roughly 40 – 50% of basket cell synapses (Foldy et al., 2010) and axons (Glickfeld and Scanziani, 2006) are located outside SP it may not be surprising that our effects span both layers. The circuit-level anatomy of CA1 is complicated, and it is certainly possible that the post-injury hyperpolarization increase has a complex dependence on location. A more parsimonious interpretation is that effects were observed in both layers because basket cells project to both layers.

A decrease in CA1 output might also contribute to the cognitive impairments associated with TBI (Cave and Squire, 1991; Kotapka et al., 1992; Asikainen et al., 1999). Although the precise functional role of cannabinoid signaling at GABAergic synapses has yet to be established, a recent report suggests that it may be especially important in mediating the interaction between the entorhinal cortex and hippocampus (Basu et al., 2013). Communication between entorhinal cortex and hippocampus is essential to temporal and spatial memory (Steffenach et al., 2005; Suh et al., 2011), and deficits in memory are a common complaint among TBI survivors (Paniak et al., 2002; Lundin et al., 2006).

There is growing appreciation for the number and diversity of interneuron subtypes in the hippocampus, and a unifying theme of such studies is that different types of interneurons play very different roles in regulating network function (Klausberger and Somogyi, 2008; Lovett-Barron et al., 2012; Varga et al., 2012). As the current study demonstrates, however, different interneurons in CA1 are likely to differ in their response to injury, and the development of effective therapeutic strategies for TBI will require a clear understanding of which cell types are affected. The current study makes important advances in that direction by identifying the locations of the altered responses in CA1, and a specific population of interneurons contributing to those changes.

## Conflict of Interest Statement

The authors declare that the research was conducted in the absence of any commercial or financial relationships that could be construed as a potential conflict of interest.

## Supplementary Material

The Supplementary Material for this article can be found online at http://www.frontiersin.org/Journal/10.3389/fncel.2014.00435/abstract

Videos S1 and S2 Excerpted video from representative slices prepared from sham (Video S1 in Supplementary Material) and injured (Video S2 in Supplementary Material) animals. Sham video recordings are for the representative slice shown in Figure 1. Each video frame represents 2 ms, and the stimulus is delivered 40 ms after the start of each video (i.e., at frame 20). Note post-injury increase in SP and SO hyperpolarization (more blue pixels) starting at approximately 30 ms after the stimulus (frame 35). The pseudocolor scale is the same as that shown for the movie frames in Figure 1. The play back speed is 15 frames per second. Videos are 178 ms excerpts from the original 750 ms recording. Stimulus: 100 μA.
